# Hands-on-training tailored in response to pre-questionnaire-based survey on image-guided brachytherapy effectively reduces anxiety about its implementation

**DOI:** 10.1093/jrr/rrae013

**Published:** 2024-03-27

**Authors:** Noriyuki Okonogi, Naoya Murakami, Jun Takatsu, Kotaro Iijima, Terufumi Kawamoto, Masaki Oshima, Kae Okuma, Koji Masui, Kazutoshi Murata, Hiroyuki Okamoto, Ken Yoshida, Shin-ei Noda, Haruko Numajiri, Miho Watanabe, Keisuke Tsuchida, Yoichi Muramoto, Tatsuki Karino, Tatsuya Ohno, Naoto Shikama

**Affiliations:** Department of Radiation Oncology, Juntendo University Graduate School of Medicine, 2-1-1 Hongo, Bunkyo-ku, Tokyo 113-8421, Japan; Department of Radiation Oncology, Juntendo University Graduate School of Medicine, 2-1-1 Hongo, Bunkyo-ku, Tokyo 113-8421, Japan; Department of Radiation Oncology, Juntendo University Graduate School of Medicine, 2-1-1 Hongo, Bunkyo-ku, Tokyo 113-8421, Japan; Department of Radiation Oncology, Juntendo University Graduate School of Medicine, 2-1-1 Hongo, Bunkyo-ku, Tokyo 113-8421, Japan; Department of Radiation Oncology, Juntendo University Graduate School of Medicine, 2-1-1 Hongo, Bunkyo-ku, Tokyo 113-8421, Japan; Department of Radiation Oncology, Juntendo University Graduate School of Medicine, 2-1-1 Hongo, Bunkyo-ku, Tokyo 113-8421, Japan; Department of Radiation Oncology, National Cancer Center Hospital, 5-1-1 Tsukiji, Chuo-ku, Tokyo 104-0045, Japan; Department of Radiology, Kyoto Prefectural University of Medicine, 465 Kajii-cho, Kawaramachi-Hirokoji, Kamigyo-ku, Kyoto 602-8566, Japan; National Institutes for Quantum Science and Technology, QST Hospital, 4-9-1 Anagawa, Inage-ku, Chiba 263-8555, Japan; Radiation Safety and Quality Assurance Division, National Cancer Center Hospital, 5-1-1 Tsukiji, Chuo-ku, Tokyo 104-0045, Japan; Department of Radiology, Kansai Medical University Medical Center, 10-15 Fumizono-cho, Moriguchi City, Osaka 570-8507, Japan; Department of Radiation Oncology, Saitama Medical University International Medical Center, 1397-1 Yamane, Hidaka City, Saitama 350-1298, Japan; Department of Radiation Oncology, Proton Medical Research Center, University of Tsukuba Hospital, 2-1-1 Amakubo, Tsukuba, Ibaraki 305-8576, Japan; Department of Diagnostic Radiology & Radiation Oncology, Chiba University Hospital, 1-8-1 Inohana, Chuo-ku, Chiba-shi, Chiba 260-8677, Japan; Department of Radiation Oncology, Kanagawa Cancer Center, 2-3-2 Nakao, Asahi-ku, Yokohama 241-8515, Japan; Department of Radiation Oncology, Juntendo University Graduate School of Medicine, 2-1-1 Hongo, Bunkyo-ku, Tokyo 113-8421, Japan; Department of Radiation Oncology, Juntendo University Graduate School of Medicine, 2-1-1 Hongo, Bunkyo-ku, Tokyo 113-8421, Japan; Department of Radiation Oncology, Gunma University Graduate School of Medicine, 3-39-22 Showa-machi, Maebashi, Gunma 371-8511, Japan; Department of Radiation Oncology, Juntendo University Graduate School of Medicine, 2-1-1 Hongo, Bunkyo-ku, Tokyo 113-8421, Japan

**Keywords:** hands-on-training, questionnaire-based survey, 3D image-guided brachytherapy, cervical cancer

## Abstract

This study assessed the significance of hands-on-training (HoT) and questionnaire-based surveys on 3D image-guided brachytherapy (3D-IGBT) and a combination of intracavitary and interstitial brachytherapy, the so-called ‘hybrid’ BT (HBT), in uterine cervical cancer. In October 2023, 29 radiation oncologists, nurses, radiologic technologists and medical physicists from 10 Japanese facilities participated in an HoT on 3D-IGBT and HBT. Questionnaires were distributed to each participant before and after the HoT, and feedback was obtained through online channels. The questionnaire response rate was 83% (24/29), with at least one participant responding from each facility. ‘Insertion of applicators and needles’, ‘human resource shortage’ and ‘pain relief and sedation’ were the primary concerns of radiation oncologists. ‘Applicator reconstruction’, ‘ optimization of dwell positions’, ‘ treatment planning’ and ‘ human resource shortages ’ were the primary concerns of radiological technologists and medical physicists. The HoT content was adjusted according to the results of preliminary surveys. The concerns expressed by the participants were addressed during the lectures and practical training. Significant reductions in anxiety were observed toward all items of the 10-point self-assessment after the HoT, regardless of the profession. The average score on satisfaction with the HoT (on a 10-point scale) was 9.52 (minimum of 8 and maximum of 10). In conclusion, HoT tailored in response to a pre-questionnaire-based survey effectively reduced participants’ anxiety regarding the implementation of 3D-IGBT and HBT.

## INTRODUCTION

Brachytherapy (BT) plays an essential role in the management of patients with uterine cervical cancer. Since the Groupe Europe de Curiethérapie and European Society for Radiotherapy and Oncology proposed the concept of 3D image-guided brachytherapy (3D-IGBT) in 2005, the uptake of this treatment method has spread rapidly worldwide [1, 2]. Recently, favorable results have been reported with the use of magnetic resonance imaging-based- or computed tomography-based 3D-IGBTs [3–6]. Hence, 3D-IGBT is now the standard of care for patients with cervical cancer.

Recently, 3D-IGBT has become increasingly sophisticated, resulting in a combination of intracavitary and interstitial BT, the so-called ‘hybrid’ BT (HBT). HBT has been found to be effective against locally advanced tumors and irregular-shaped tumors [6–8]. HBT was performed in 43.0% of patients according to a recent prospective study [6], indicating that this technique is an essential part of clinical practice.

Effective multidisciplinary education for all healthcare professionals involved in the BT process is critical for successful implementation of 3D-IGBT, particularly HBT [9]. The current options for educating professionals on the use of 3D-IGBT vary in their content [10, 11]. Hands-on-training (HoT) is beneficial for facilities that are introducing HBT for the first time or for improving their techniques because it allows the professionals to learn the procedures directly. The Japanese Society for Radiation Oncology (JASTRO) recommends widespread use of 3D-IGBT and HBT for patients with cervical cancer. The JASTRO established consensus guidelines for HBT for gynecological cancers in 2021 [12] and started HoT for HBT since 2022 [13]. Using this framework, we conducted the second HoT for 3D-IGBT, including HBT, this year, with emphasis on hands-on proficiency.

The concept of HoT and workshops in radiation oncology is not new, and their content and quality are important. To make HoT truly meaningful, it is important to understand in advance what the participants want to learn. It is also important to quantify to what extent participation in the HoT reduces anxiety about implementing HBT. In this regard, we conducted questionnaire-based surveys before and after the HoT. In this study, we report the results of the surveys and briefly discuss the significance of HoT and questionnaire-based surveys.

## MATERIALS AND METHODS

The second HoT for 3D-IGBT, including the HBT, was conducted in October 2023. A total of 29 radiation oncologists, nurses, radiologic technologists and medical physicists from 10 Japanese facilities participated in the HoT. Questionnaires were distributed to the participants before and after the HoT, and feedback was obtained through online channels. The pre-questionnaire was distributed 1 week before the HoT. The post-questionnaire was distributed on the day of the HoT, and responses were collected 2 weeks later.

The questionnaire was administered to radiation oncologists, radiologic technologists, medical physicists and nurses and consisted of questions on their concerns about performing HBT. The questionnaire also asked the respondents to rate their level of concern with each HBT procedure on a scale of 1 to 10. In the post-questionnaire survey, the respondents were asked to rate their satisfaction with the HoT on a scale of 1 to 10 and their willingness to proceed with HBT when they encountered patients who may be candidates for it in the future. Details of the pre- and post-course questionnaires are provided in the Supplementary Data 1. Changes in the degree of anxiety before and after the HoT were evaluated using paired-samples *t*-test. All statistical tests were two-sided, with *P* < 0.05 designated as the level of statistical significance. Excel (Microsoft, Redmond, WA) for Mac (2021) was used to perform statistical calculations.

## RESULTS AND DISCUSSION

The questionnaire response rate was 83% (24/29), with at least one participant responding to each facility. In all, 11 radiation oncologists, 1 nurse, 12 radiologic technologists and medical physicists responded. Regarding the periods in which they had been engaged in gynecological BT, 10 had worked for <1 year, 3, for between 1 and 5 years, and 11, for more than 5 years.

**Fig. 1:**
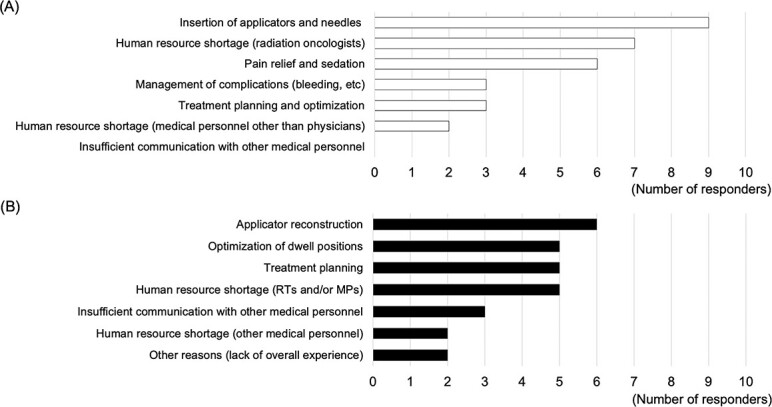
Concerns in performing HBT. (**A**) Concerns expressed by radiation oncologists in the pre-training questionnaire, with up to three items per person. (**B**) Concerns expressed by radiologic technologists and medical physicists in the pre-training questionnaire, with up to three items per person. RT = radiologic technologist, MP = medical physicist.

Figure 1 shows the concerns regarding the performance of HBT obtained from the pre-course questionnaire. ‘Insertion of applicators and needles’, ‘human resource shortage’ and ‘pain relief and sedation’ were the primary concerns of radiation oncologists. ‘Applicator reconstruction’, ‘optimization of dwell positions’, ‘treatment planning’ and ‘human resource shortage’ were the primary concerns of radiological technologists and medical physicists. ‘Human resource shortage’ was the primary concern of one nurse who participated in the HoT.

The HoT content was adjusted according to the results of preliminary surveys. The concerns expressed by the participants were addressed in the lectures and practical training. Specifically, participants’ concerns were shared in advance with the instructors. Then, following changes were made:

(1) A workshop was organized in which experts answered basic questions from participants.
(2) Applicator insertion videos were made and presented.
(3) Extended time for medical physicists to explain and demonstrate the tips of the applicator reconstruction.
(4) A lecture and demonstration of a sacral block as a pain relief technique (including hand-made phantoms for the procedure) were provided.

**Fig. 2:**
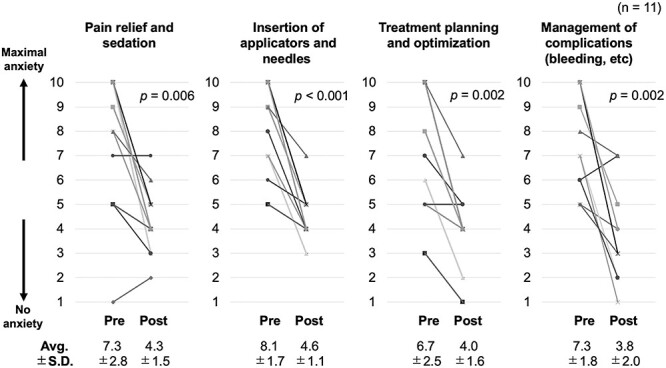
Changes in the quantitative responses of participants (radiation oncologists) before and after the course. Each graph shows the change in degree of anxiety for each item, with 10 indicating maximum anxiety and 1 indicating no anxiety. Avg. = average, SD = standard deviation.

**Fig. 3:**
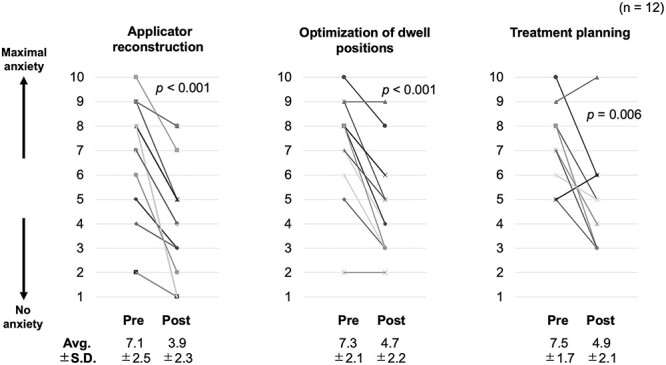
Changes in the quantitative responses of participants (radiologic technologists and medical physicists) before and after the course. Each graph shows the change in degree of anxiety for each item, with 10 indicating maximum anxiety and 1 indicating no anxiety. Avg. = average, SD = standard deviation.

Figure 2 shows the changes in the quantitative responses of participants (radiation oncologists) before and after the course. Significant reductions in anxiety were observed for all items of the 10-point self-assessment of the HoT. In particular, a significant reduction in anxiety was observed with regard to ‘insertion of applicators and needles’ (*P* < 0.001). Figure 3 shows the changes in the participants’ quantitative responses (radiologic technologists and medical physicists) before and after the course. Similar to the results of radiation oncologists, radiologic technologists and medical physicists showed significant reductions in anxiety toward all items in the 10-point self-assessment of the HoT. All respondents showed a reduction in anxiety about ‘applicator reconstruction’ (*P* < 0.001). Notably, some participants’ anxiety increased after their participation in the HoT.

The average score on satisfaction with the HoT (on a 10-point scale) was 9.52 (with a minimum of 8 and maximum of 10). The average score on willingness to perform HBT when the participants saw patients who had indications for HBT in the future was 2.10 (1 = definitely yes, 10 = no confidence). With the advancements in 3D-IGBT, HoT and other guidelines have become essential for successful implementation. Recent studies have suggested that theoretical teaching can only partially replace on-site education [14, 15]. Although online learning offers convenience, it has disadvantages such as lack of physical interaction, inability to control the audience’s environment, lack of kinesthetic learning, network interruptions, software incompatibility and hardware malfunctions [16, 17]. Online learning and HoT are complementary. Our study highlights the importance of understanding the participants’ needs in advance to maximize the significance of HoT.

To respond flexibly to the demands of the participants in the HoT, the human resources involved in the provision of the HoT must be of high quality. For our HoT, we were able to gather abundant human resources with the support of the participating institutions and JASTRO. In addition, vendor support is essential to provide practical training using equipment that can be used in actual clinical practice. In one study, a vendor reported that educational initiatives and collaboration between academia and industry are critical for well-developed HoT implementation [18].

Significant reductions in anxiety were observed in all items of the 10-point self-assessment by participating in the HoT, regardless of the profession. However, some participants’ anxiety increased after their participation in the HoT, possibly because of reaffirmation of their own HBT abilities in light of the new skills and knowledge gained through the HoT. A recent BT training survey conducted among radiation oncology residents in Europe suggested the need for a curriculum with easy access to trained instructors [19]. Taken together, systematic and comprehensive follow-up is required for HBT to take root.

A limitation of this study is that it analyzed the results of a survey of a small number of professionals who underwent a single HoT. Furthermore, the true endpoint of the HoT is not participant satisfaction or improved skills and knowledge, but rather the actual provision of HBT to the patients at the participant’s facility. It is critical to provide long-term support to the participants in this HoT to determine whether they can successfully implement HBT.

In conclusion, HoT tailored in response to a pre-questionnaire-based survey effectively reduced participants’ anxiety regarding the implementation of 3D-IGBT and HBT. We hope that through activities such as HoT, more facilities will be able to provide 3D-IGBT and HBT and that high-quality radiation therapy for cervical cancer will become more widely available.

## CONFLICT OF INTEREST

Dr Murakami, Dr Okonogi and Dr Shikama report grants from Elekta KK, Chiyoda Technol Co. and Boston Scientific.

## FUNDING

This study was supported by a Grant-in-Aid for Scientific Research (22K07779, 23H02869, and 23K07171) from the Ministry of Education, Culture, Sports, Science and Technology of Japan.

## PRESENTATION AT A CONFERENCE

None.

## Supplementary Material

Suppl_rrae013
