# Supplementary material for: Hands-on-training tailored in response to pre-questionnaire-based survey on image-guided brachytherapy effectively reduces anxiety about its implementation
Source: J Radiat Res. 2024 Mar 27;65(3):323–7. doi: 10.1093/jrr/rrae013 (PMC11115464; doi:10.1093/jrr/rrae013)
Supplement: Suppl_rrae013 [file suppl_rrae013.docx]

**Supplementary Data: Pre and post course questionnaires.**

**(A) Pre course questionnaire**

- [To ALL] How long have you been engaged in brachytherapy for gynecological tumors (please choose the closest one)?
  - Little or nothing
  - Less than six months
  - More than six months but less than one year
  - More than one year but less than three years
  - More than three years but less than five years
  - More than 5 years
- [To radiation oncologists] Please rate your concern about hybrid brachytherapy at your facility from 1 to 10 (1: No anxiety 10: Maximum anxiety).
  - Pain relief and sedation
  - Insertion of applicators and needles
  - Treatment planning and optimization
  - Management of complications (bleeding, etc)
- [To radiation oncologists] Please select the barriers to implementing hybrid irradiation from the list below (up to three items).
  - Human resource shortage (radiation oncologists)
  - Human resource shortage (medical personnel other than physicians)
  - Insertion of applicators and needles
  - Insufficient communication with other medical personnel
  - Management of complications (bleeding, etc.)
  - Pain relief and sedation
  - Treatment planning and optimization
  - Other reasons (feel free to write down)
- [To radiologic technologists and medical physicists] Please rate your concern about hybrid brachytherapy at your facility from 1 to 10 (1: No anxiety 10: Maximum anxiety).
  - Applicator reconstruction
  - Optimization of dwell positions
  - Treatment planning
- [To radiologic technologists and medical physicists] Please select the barriers to implementing hybrid irradiation from the list below (up to three items).
  - Applicator reconstruction
  - Human resource shortage (RTs and/or MPs)
  - Human resource shortage (other medical personnel)
  - Insufficient communication with other medical personnel
  - Optimization of dwell positions
  - Treatment planning
  - Other reasons (feel free to write down)
- [To nurses] Please rate your concern about hybrid brachytherapy at your facility from 1 to 10 (1: No anxiety 10: Maximum anxiety).
  - Pain relief and sedation
  - Management of complications (bleeding, etc.)
  - Understanding the overall treatment
- [To nurses] Please select the barriers to implementing hybrid irradiation from the list below (up to three items).
  - Human resource shortage (nurses)
  - Human resource shortage (medical personnel other than nurses)
  - Insufficient communication with other medical personnel
  - Management of complications (bleeding, etc.)
  - Pain relief and sedation
  - Understanding the overall treatment
  - Other reasons (feel free to write down)

**(B) Post course questionnaire**

* Same questions as in Pre course questionnaire for comparison

- [To ALL] Please rate your satisfaction with this seminar from 1 to 10 (1: Poor, 10: Excellent)
- *[To radiation oncologists] Please rate your concern about hybrid brachytherapy at your facility from 1 to 10 (1: No anxiety 10: Maximum anxiety).
  - Pain relief and sedation
  - Insertion of applicators and needles
  - Treatment planning and optimization
  - Management of complications (bleeding, etc)
- Would you recommend hybrid brachytherapy in future if a patient comes to you with the indication?　Please answer on a scale of 1 to 10 (1: Definitely yes, 10: No confidence).
- *[To radiologic technologists and medical physicists] Please rate your concern about hybrid brachytherapy at your facility from 1 to 10 (1: No anxiety 10: Maximum anxiety).
  - Applicator reconstruction
  - Optimization of dwell positions
  - Treatment planning
- *[To nurses] Please rate your concern about hybrid brachytherapy at your facility from 1 to 10 (1: No anxiety 10: Maximum anxiety).
  - Pain relief and sedation
  - Management of complications (bleeding, etc.)
  - Understanding the overall treatment
- Feel free to write down.

//
